# "We are survivors and not a virus:" Content analysis of media reporting on Ebola survivors in Liberia

**DOI:** 10.1371/journal.pntd.0005845

**Published:** 2017-08-24

**Authors:** Elisabeth Anne-Sophie Mayrhuber, Thomas Niederkrotenthaler, Ruth Kutalek

**Affiliations:** 1 Unit Medical Anthropology and Global Health, Department of Social and Preventive Medicine, Center for Public Health, Medical University of Vienna, Kinderspitalgasse 15, Vienna, Austria; 2 Unit Suicide Research, Department of Social and Preventive Medicine, Center for Public Health, Medical University of Vienna, Kinderspitalgasse 15, Vienna, Austria; Institute for Disease Modeling, UNITED STATES

## Abstract

**Background:**

The Ebola virus disease epidemic between 2013 and 2016 in West Africa was unprecedented. It resulted in approximately 28.000 cases and 10.000 Ebola survivors. Many survivors face social, economic and health-related predicaments and media reporting is crucially important in infectious disease outbreaks. However, there is little research on reporting of the social situation of Ebola survivors in Liberia.

**Methods:**

The study used a mixed methods approach and analysed media reports from the Liberian Daily Observer (DOL), a daily newspaper available online in English. We were interested to know how the situation of Ebola survivors was portrayed; in what way issues such as stigma and discrimination were addressed; and which stigma reduction interventions were covered and how. We included all articles on the situation of Ebola survivors in the quantitative and in-depth qualitative analysis published between April 2014 and March 2016.

**Results:**

The DOL published 148 articles that portrayed the social situation of Ebola survivors between the 24 months observation period. In these articles, Ebola survivors were often defined beyond biological terms, reflecting on a broader social definition of survivorship. Survivorship was associated with challenges such as suffering from after-effects, social and economic consequences and psychological distress. Almost 50% of the articles explicitly mentioned stigmatisation in their reporting on Ebola survivors. This was contextualised in untrustworthiness towards international responses and the local health care system and inconclusive knowledge on cures and transmission routes. In the majority of DOL articles stigma reduction and engaging survivors in the response was reported as crucially important.

**Discussion:**

Reporting in the DOL was educational-didactical and well-balanced in terms of disseminating available medical knowledge and reflecting the social situation of Ebola survivors. While the articles contextualised factors contributing to stigmatisation throughout the reporting, journalistic scrutiny regarding effectiveness of interventions by government and NGOs was missing.

## Author summary

The largest Ebola epidemic occurred in West Africa between 2013 and 2016. Liberia was one of the most affected countries with more than 1500 survivors registered. In the height of the Ebola outbreak survivors were increasingly included in the treatment and care of patients and in health sensitizing and social mobilisation efforts. However, the reintegration of survivors back into their communities proved to be challenging across West Africa. Media reporting plays a crucial role in health emergency situations. It gives visibility to what is considered as relevant, frames risk perception and can induce positive health behaviour practices and attitudes. In this study we analysed how one of Liberia’s largest newspapers portrayed and informed the public on the social situation of survivors, in what way it addressed the issues of stigma and discrimination and which stigma reduction interventions were covered and how. We found that reporting was overall comprehensive and well-balanced in terms of disseminating available medical knowledge, scrutinizing stigma. Reports also reflected on contributing factors such as untrustworthiness towards response as well as inconclusive understanding of cures and transmission routes. In a larger context this specific reporting was acting as corrective to what the larger public believed.

## Introduction

The Ebola virus disease epidemic in West Africa 2013–2016 was unprecedented and resulted in over 28.000 cases. Up to 10.000 persons are estimated to have survived the disease [1]. In Liberia, there are over 1500 survivors registered in the database of the Liberian Ministry of Health [2] but considering the high overall WHO estimates it is assumed that the actual number of survivors is significantly higher than the ones registered.

During the height of the epidemic an important component of a comprehensive disease response was to include survivors in the treatment and care of EVD patients, in training of health workers, as well as in health sensitizing and social mobilization efforts [3–5]. However, the reintegration of survivors into their communities proved to be challenging. While at the beginning of the epidemic survivors were often welcomed by their communities [6], EVD-related stigma that led to social isolation of survivors in families and communities, physical violence and loss of jobs has been reported from the affected countries [3, 7–9]. Stigma affects the social lives of individuals and communities, leading to suffering and loss of important networks. Stigma and social distancing is also associated with avoidance of seeking health care [10, 11] which in the case of Ebola virus disease is important regarding isolation and treatment of sick persons, and quarantine of contacts. Stigmatization of survivors in the health system might also lead to neglect or survivors not disclosing their status. Survivors have lost their possessions to prevent disease transmission and they often suffered income loss. Moreover, many are suffering from physical after-effects of the disease [12], as well as mental and psychological consequences [8, 13–16].

Considering the high numbers of survivors in this latest epidemic there is little research on the social situation of survivors in the most affected countries [17]. In this study we therefore wanted to analyse how one of Liberia’s largest newspapers, the Liberian Observer, portrayed and informed the public on the situation of Ebola survivors 2014–2016. We were interested in who is considered a survivor, the challenges survivors face in day-to-day life, and how survival was explained and disease transmission linked to survivors. Furthermore, we were interested how the discourse on stigma and discrimination was conveyed to the readership. Stigmatization of survivors has recently received heightened attention through the tragic and unnecessary death of a female survivor who was not treated after complications in childbirth [18]. Moreover, we wanted to understand how these themes changed over time–from the beginning of the epidemic in March 2014, through the peak in December 2014, to its consolidation in 2015 and up to March 2016 when the epidemic waned.

There are ongoing discussions over who is to be considered a survivor of EVD. This question is important because 1) survivors are eligible for social and medical support, 2) survivors are often part of clinical studies, and 3) the identity of survivorship is important when discussing the social implications of the epidemic. One of the first agreed upon biological definitions of survivorship in Liberia was discussed at a meeting of the Liberian Ministry of Health and Social Welfare. The group came to the consensus that a survivor should be defined as “A person who tested positive for the Ebola virus disease and after receiving care and treatment, recovered and tested negative”[19]. However, one of the problems is the often poor record keeping in facilities that makes certification of survivors difficult [19, 20]. Another question was what should be done with persons who were sick and survived but were not treated in a facility and thus not tested? Furthermore, towards the end of the epidemic it became clear, that minimally symptomatic Ebola virus infections were likely to have taken place [21] and the question arose “Should the notion of survivorship be extended to all those who are IgG positive, including those who had minimally symptomatic infection or who were sick but were never tested at the time of illness?” [21]. Thus WHO [1] defines a survivor as a person “With a confirmed positive result on RT-PCR testing for Ebola virus on any body fluid who subsequently recovered; and/or who is IgM and/or IgG positive on serological testing for EVD and has not been vaccinated against Ebola virus.” This definition, however, does not answer the social and economic implications of survivorship.

This study contributes to the research field of media reporting in public health emergencies. Media reporting is crucially important in infectious disease outbreaks. One study, for instance, found an association between media reports of interventions that provided education and reductions in transmissions of Ebola in Sierra Leone and Liberia [22]. Real-time information from trustworthy news reports have been used to characterize epidemiological patterns [23]. Media can be equally important allies for raising awareness, inducing positive healthy behaviour practice [24] and disseminating vital information in emergencies. Media reporting gives visibility to what is seen as important, it frames risk perceptions and allows reflection of public health policy such as promoting necessary intervention strategies. It can also be useful in promoting behaviour change and mitigating stigmatization [25–27]. However, any form of communication in public health emergencies has to consider people’s priorities as well as social, political, economic and historic contexts [28, 29].

Several studies have dealt with the role of media in the Ebola epidemic in West Africa, most of them focusing on the situation in the global north, especially the U.S. [30–35]. Only few studies investigate media reporting on Ebola in the affected countries themselves. Two studies looked at how the Ebola crisis was framed by newspapers from the US and Canada, compared to an “insider” perspective of an Ebola affected country (Sierra Leone and Nigeria) [36, 37]; one assessed the role of four Nigerian newspapers in creating awareness to stop the spread of Ebola [38]. To our knowledge, however, there is no study on media reporting in the Ebola epidemic in Liberia and no research so far has been published on the portrayal of Ebola survivors in the media during the epidemic in West Africa.

The main research questions are therefore: How is the situation of Ebola survivors portrayed in one of Liberia’s largest newspapers? In what way are issues such as stigma and discrimination against Ebola survivors addressed and what factors are identified to contribute to it? Which stigma reduction interventions are covered and how?

According to Peacebuilding-Data newspapers are consumed at least occasionally by one third of the population in Liberia (29%). The most commonly read newspaper is the Daily Observer (21%), followed by the Inquirer (11%) and the New Democrat (10%) [39]. The Daily Observer is available in English which is the country’s only official language. Besides varieties of Liberian English, there are over 30 other languages spoken in the country. A total of 47,6% of the population is literate [40] and it can be assumed that the Daily Observer addresses a distinct, educated readership rather than the general population.

## Material and methods

### Design

This study used a mixed method approach, whereby the Liberian Daily Observer (DOL) a daily newspaper released in English was analysed. It is available online (www.liberianobserver.com) and is reportedly the largest Liberian newspaper [41]. All articles that were published in the DOL on the situation of Ebola survivors between April 2014 and March 2016 were collected through the archive and search function. We used the following simple search terms: “Ebola survivors”, “Ebola”, “survivors” and “survive”, screened the articles applying inclusion and exclusion criteria. We performed a basic quantitative content analysis [42] and an in-depth qualitative analysis [43, 44], guided by the research question.

### Inclusion and exclusion criteria of articles

Using the above mentioned search terms EM initially collected 630 articles and screened the articles for abstract and titles. Articles from all rubrics were principally included. The article had to report on any aspect of Ebola survivorship–challenges faced, after effects, and survivors as factors of EVD transmission. Articles were excluded if they only reported about survivors (of other disease) but not in regard to Ebola, if they only mentioned Ebola survivors as receiver group of donations but lacked any further or contextual information, or if they had fictional character such as short stories or poetry. The inclusion and exclusion criteria were firstly discussed in the research team (EM, RK, TN) and consecutively applied by EM resulting in 148 final articles for analysis.

### Data analysis

The selected articles were imported into the Qualitative Data Analysis Software atlas.ti in word or pdf format and were analysed [45]. Two of the authors (EM, RK) developed the coding framework. First, all collected articles were reviewed by EM and main themes were identified following an open inductive coding approach [45]. In discussion with RK who has worked with Ebola survivors in Liberia, EM compared these main themes, further categorized and supplemented them with sub-themes. The data was then quantitatively analysed; in a first step the frequency of publication of articles per month was recorded, and descriptive information such as date of publication and type of article was registered (EM) [42]. This was followed by an in-depth qualitative analysis to investigate which themes were addressed and how the situation of Ebola survivors was portrayed in the newspaper [43]. In accordance to the main research questions, we looked at how survivors were defined, what and how challenges of living as survivor were described, and how the themes stigma and discrimination were presented. Related themes to codes that emerged in open coding through re-reviewing the articles were noted and adapted with deductive categories that were discussed among all three authors after one third of the articles were coded in atlas.ti. The following codes were used: personal story/situation and release, health care workers, after-effects, how/why survived, transmission through survivors, stigma explicit, stigma implicit, valuable involvement of survivors, support received, identity, what is lost (for an overview of codes see Table 1). We assessed inter-coder reliability, or more specifically inter-coder agreement, using Cohen’s kappa technique [46, 47]. RK coded twenty articles independently (14% of articles chosen randomly from the sample) and agreement between 0.67 and 1.00 was reached between the two coders EM (coder 1) and RK (coder 2), see Table 1. Codes not applied to the 20 articles are not appearing in the table. In accordance to Landis and Koch (1977) strength of agreement division for Kappa Statistics (<0.00 Poor; 0.00–0.20 Slight; 0.21–0.40 Fair; 0.41–0.60 Moderate; 0.61–0.80 Substantial; 0.81-1-00 Almost Perfect) it lies between substantial and almost perfect [48].

**Table 1:** Kappa strength of agreement.

Code	Coder 1	Coder 2	Exact match	Kappa
personal story/ situation and release	4	6	4	0.67
After effect	8	8	8	1.00
Transmission through survivors	20	19	19	0.95
Stigma explicit	17	19	17	0.89
Stigma implicit	6	4	4	0.67
Valuable involvement survivors	4	4	3	0.60
Support received	13	10	10	0.77
What is lost	2	3	2	0.67
Totals	74	73	67	0.78

### Ethical approval

This media analysis was conducted with already published newspaper articles; therefore, no ethical approval was necessary.

## Results

Of the selected 148 articles, 64 were from 2014 (A1-A64), 77 from 2015 (B1-B77) and 7 from 2016 (C1-C7).

**Fig 1:**
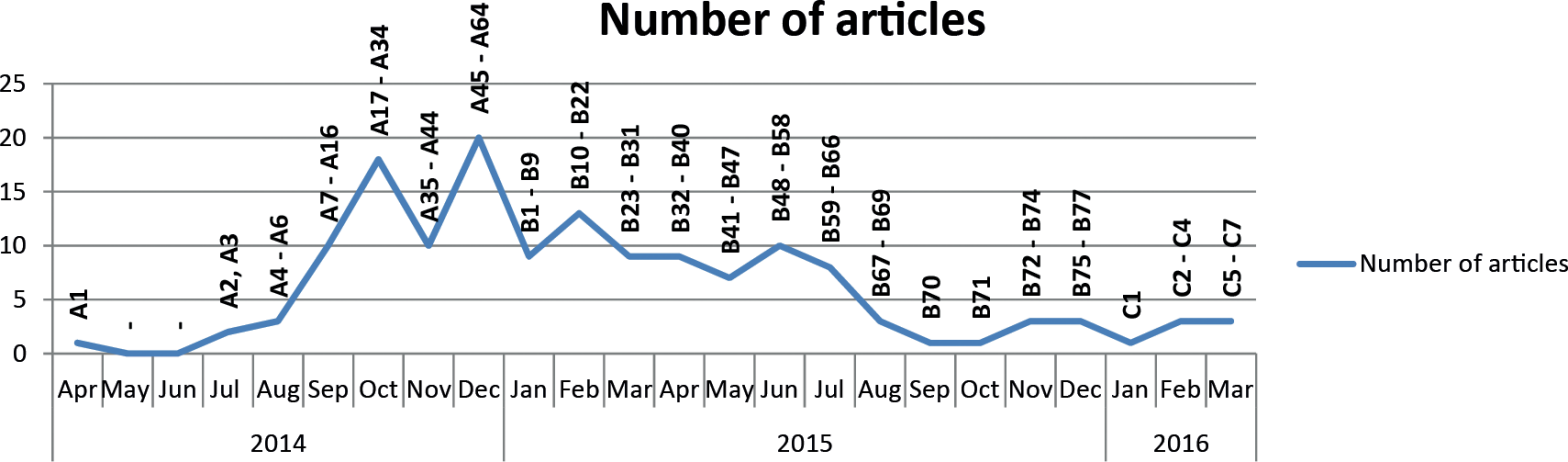
Number of articles published per month between April 2014 and March 2016.

The frequency of published articles on the specific situation of survivors varied over the 2-year observation period. While between April to August 2014, and August 2015 to March 2016, less than four articles were published per month, between September 2014 to July 2015 on average of 11 articles per month were published, with a peak in December 2014 of 20 articles see Fig 1.

Regarding the type of publication, we found 61% of the articles were published under the news rubric, 26% under a columns rubric, whereas very few articles were published under sports (5%), opinion commentary (3%) or business (3%) rubric see Table 2.

**Table 2:** Type of publication in the daily observer Liberia.

Type of article	n	%
	148	100
News	91	61
Column *(education*, *health*, *women*, *youth)*	38	26
Sports	8	5
Opinion commentary	4	3
Business	5	3
no assignment	2	1

### Complex representation of survivors

The DOL referred to two groups as survivors: persons who were confirmed Ebola positive cases who later tested negative; and persons who were not infected themselves but were closely associated to the disease, e.g. who lost partners and family members, orphans, persons discharged from ETUs without being infected or persons who “survived” quarantine (A1-A64, B1-B77, C1-C7). The representation of survivors used by the DOL is assumed to have originated from how people in Liberia were using the term Ebola survivor. Some articles also referred to survivors as “Ebola victims” (A6, A12, A19, A50, A52, B8, B22, B31, B51, B63, B64, C3) or “viral victims” (B3). Campaigns against stigmatization often targeted “Ebola victims & survivors” or “Ebola affected children/persons and survivors”, highlighting that psychosocial support was pivotal for survivors themselves but also for their families, friends and communities (e.g. A19, A27, A45, A46, A47, A52, A55, B1, B3, B8, B9, B11, B39, B41, B63, B65, B66). The complex representation of who is a survivor is linked to the fact that both survivors and victims are rather classified by their marginalization and affectedness by the disease than by biological survival alone. Survivorship per se was associated with support by various programs. However, this identity might change in the course of time, as one business woman who offered training to survivors is cited:

“They will not always be called Ebola survivors so it is now time for them to make use of what they are getting from donor organizations to empower themselves’ […] Ebola survivors are now receiving little funding from organizations of goodwill […] their title as “Ebola Survivors” would eventually change as time goes by.” (B71, 10/06/2015)

While being called an “Ebola survivor” by others is often felt stigmatizing, the term “survivor” might turn into a positive identity if used by the ones affected.

“Instead of using my name, they were calling me ‘Ebola survivor’. […] I was so depressed I came really close to killing myself. But I survive. I am a survivor after all” (B12, 02/09/2015)

The denomination of survivor becomes also important when highlighting the fact that survivors are immune to the disease. This was used in several awareness campaigns:

“Normally, when you are pronounced [an] Ebola survivor or patient, it stigmatizes you. But since we are getting to understand what Ebola is and how it works and what are the preventive measures, so we thought it wise that we cannot get the Ebola from them when they have already been pronounced survivors” (B77, 12/27/2015)

Over the course of the epidemic the DOL increasingly cited Ebola survivors as heroes and heroines. Already in November 2014 two discharged health care workers were referred to as “healthcare heroes” (A49) and also later discharged health care workers were described as the “true heroes and heroines” (B17), or heroic icons who cared for the infected (B6, C1). General views that survivors should be viewed as heroes are also found in some of the articles (B3, B24, B46). The country director of an NGO for instance explained: “[…] that survivors, mainly orphans, should be taken care of and embraced in the society because they are heroes of the Ebola crisis” (A55).

### Situation of survivors

The DOL reports that Ebola survivors face a multitude of health related, economic and social difficulties and were furthermore subject to discrimination and stigma during and in the aftermath of the epidemic.

Eighteen articles (12%) address the issue of health related after-effects of Ebola survivors which included visual problems or blindness, headaches, hearing impairment, joint-, muscle- and back pain, weakness, breathlessness, diarrhoea, abdominal pain, changes in the immune system, fever, swollen legs, loss of appetite, impotency or uncontrollable erection for men and failure to conceive or get pregnant by women (A14, A24, A59, B3, B34, B49, B60, B65, C2, C4). The articles also report on neurological symptoms, psychological distress and mental health issues (A64, B9, B34, B60, B61, B76). The reporting of sequelae increased in frequency when the PREVAIL Study lll, which is a Liberia-U.S. clinical research partnership on Ebola survivors to study long-term health effects of EVD, was launched and recruitment of study participants apparently started. One survivor explained her feeling of overall powerlessness:

“[…] since I left the ETU, I have been experiencing one problem after another. Ebola made me a cripple. I can’t even put on my own clothes by myself except someone helps me.” (B60, 07/07/15)

Another study participant describes his psychological problems:

“I have developed so many psychological problems since I left the Ebola Treatment Unit. Some of those problems are occasional mental illness. I sometimes get angry without remembering afterward. I am very forgetful again.” (B60, 07/07/2015)

Social and economic consequences of the disease were portrayed in the DOL especially with regards to survivors’ experiences of discrimination and stigmatization. In this context the problematic situation of orphans who survived was also highlighted in several articles (A27, A52, A53, A55, A56, A61, A64, B8, B11, B15, B31, B34, B37, B39, B41, B50, B51, C5). The overall situation of survivors is portrayed as severely distressing; several articles refer to the loss of children and family members due to the disease (A7, A26, A27, A30, A31, A43, A44, A46, A47, A48, A50, A51, A53, A56, A64, B7, B13, B15, B22, B34, B37, B46, B60, B68, B72), the loss of hope (A3, A28, B68) and a situation of despair. One survivor explains:

“I lost all of my people. I lost my father and mother and all of my brothers and sisters. As of now, I have no immediate relatives. I’m an orphan now and have no means to sustain myself as I have lost everything. Sometimes I sit and cry endlessly. It is very difficult to understand.” (A27, 10/23/2014)

Loss also included “capacity to sustain herself […] and thinking about nothing except death” (A38) and lost sources of income (A44, A53) “[…] and every other hope of livelihood has been lost” (A27). A female survivor describes the difficulty to sustain normal life:

“We have some people that cannot function; some are suffering from eyesight problems and many need medical attention but don’t have the money for further treatment. Some of us lost all our supporters” (B5, 01/14/2015)

Also in terms of material losses DOL articles reported that household items, beddings and other belongings were burned:

“[…] those who survived the EVD had lost everything they ever owned because community members feared that keeping such possessions would have further spread the virus.” (A60, 12/22/2014)

### Ebola related stigma and discrimination

Almost fifty percent of the articles (74 articles) explicitly mention “stigmatization” or “stigma” in their reporting on Ebola survivors. While in 2014 only 34% articles mention stigma or stigmatization the number increases in 2015 to 64% and in 2016 to 43%. Of those 74 articles which explicitly mention stigma or stigmatization in their reporting on Ebola survivors, 50% (37 articles) were published in the news section, 30% (22 articles) in the column section (including education, health, women and youth), 11% (8 articles) in the sports section, three articles mentioning stigma or stigmatization occurred in the business section and two articles respectively as opinion commentary or could not be assigned.

“[…] survivors have complained of being stigmatized and discriminated against […] people who have come in contact with infected persons, even their families, even though declared free of the disease, are stigmatized.” (A63, 12/31/2014)

“Ebola survivors in the country had largely been abandoned by family members and friends, leaving them vulnerable and stigmatized at all levels” (A61, 12/22/2014)

The articles often describe that stigmatization resulting in discrimination was experienced as a community attitude and resentment and ostracism was often expressed by shunning and avoiding survivors or other persons associated with the disease (A25, B32, B77). Articles also describe survivors in a situation where they experience isolation (A49) and where they have nowhere to go (A64) and desperation on their situation was linked to mental health issues (see above). Other acts of discrimination referred to in the DOL spanned from evictions from home, joblessness, separation from community and family members to acts of violence, such as forced prohibition to use community wells (A51). Some articles reported on stigma leading to discrimination at the work place, which had devastating economic effects and often result in aid dependency (A51, B34, B44, C5). Another aspect reported on was that many Liberians refuse Ebola survivors as tenants (A47, A49, A59, C5), sometimes leading to eviction and homelessness (A59, B12).

“My landlord issued an eviction notice. She was blaming me because the authorities had imposed quarantine on her house I was living and after burning all my belongings. I asked the elderly in my community for help but she would not listen to them and increased the rental fee so I could not afford it anymore.” (B12, 02/09/2015)

It was reported that survivors also had problems buying food because marketers refused to accept money from them (A31, B12, B41). Furthermore, DOL articles point to stigma and fear of patients who recovered from Ebola when seeking health care in non-Ebola healthcare structures (B40, B44), which is specifically problematic in the face of the after-effects survivors often experience. One article reports that female survivors suffer the burden of social stigmatization more than man due to their traditional role as care givers (B50).

### Factors contributing to stigma and discrimination

Several articles stressed the problem of failing communication between sick relatives that were treated in Ebola Treatment Units (ETUs) and their families and communities, as factors reinforcing fear. It was reported that for a number of days, there was no communication with patients; from a patient’s wife perspective an article narrates: “no one on the outside was telling her anything about his wellbeing even if she asked” (A5). Explanations for stigmatizing behaviour were also viewed in the context of a general untrustworthiness towards the international health response and their local establishments, the health care system as well as estimated casualty numbers, noted in some articles (A8, A29, A32, A44, A50, A62).

Many articles identified the fear of contracting the disease as the main underlying reason for any form of stigmatization and/or discrimination. The DOL cited a WHO employee, explaining:

“[…] some of the Ebola survivors who talked to her indicated that most Liberians are refusing Ebola survivor tenants for fear that they [note: owners of the flat] and their families might contract the disease” (A49, 12/11/2014)

Other articles also reported fear of contracting the disease as a contributing factor for rejection, stigma and discrimination of survivors and their families by their relatives or communities (A51, A63). In this context, several articles cite explanations for survival, e.g. early treatment and special care (A6, A9, A11, A12, A15, A23, A44, A54), other articles attribute survival to God and miracles (A2, A3, A5, A11, A17, A19, A28, A31, A38, A55). From the beginning of 2015 onwards no direct explanations for survival were portrayed in the newspaper articles any longer (B1-B77, C1-C7). Many of the DOL articles point to fear of transmission in the view of inconclusive knowledge or rather absence of cures.

“[…] it is a known fact that surviving the Ebola virus disease has to be by sheer miracle, as the disease has no known cure at the moment and it has a 90 percent case fatality rate.” (A2, 07/08/2014)

“There is no yet known cure for the virus, thus experiments should not be dismissed but subjected to further research. Donors should direct funding to research to ensure donations are not just directed at paying experts but to find a remedy.” (A32, 10/29/2014)

“[…] the Ministry of Health announced that there is no cure for the virus” (B33, 04/05/2015)

After May 2015 the DOL material did not again point to the fact that there is “no cure” or secure healing method for Ebola. Yet, another major theme that emerged from the articles was the uncertainty about possible transmission routes. Already in one of the first selected article from April 2014 the Ministry of Health was cited to encourage the public to take the following precautionary measures:

“1. Not to eat bush animals and to be very careful when handling fresh bush meat;

2. To avoid bathing dead bodies of suspected patients;

3. To avoid eating plums/mangoes and other fruits that have been partially eaten by bats;

4. To avoid coming in direct contact with body fluids of infected persons or dead persons” (A1, 04/25/2014)

Until the end of the observation period the second and fourth point became central in the debates and reports around transmission routes. Transmission became increasingly linked to biological survivors. While in 2014 only six articles reported a possible transmission through survivors (A11, A35, A36, A42, A45, A58), the frequency increased in 2015 where 18 articles linked transmission to survivors (B26, B28, B29, B30, B33, B35, B36, B38, B43, B45, B54, B57, B62, B69, B70, B73, B74, B75) and in 2016 with two articles (C1, C7). In February 2015 an article still stated that:

„[…] those who came down with the virus cannot pass it on to others once they are declared free and certificated by the country’s health authorities.”(B10)

In March 25^th^ 2015 the first EVD confirmed case was reported where transmission routes initially remained undetermined but in which a survivor was mentioned as a possible source of infection:

“The brother explained to us that Ruth recently had a dispute with her brothers, who live abroad and that’s what caused her to get sick. But she also explained to us that she came in contact with a survivor as well.” (B29, 03/25/2015)

The patient passed away on March 27^th^ and three days afterwards on March 30^th^ 2015 the headline read: “‘Sexual Transmission’ May Have Infected Ebola Patient” and the article reports:

“[…] this [sexual transmission] is far from proven as more tests are still being conducted in order to come up with the full conclusion” (B30, 03/30/2015).

**Fig 2:**
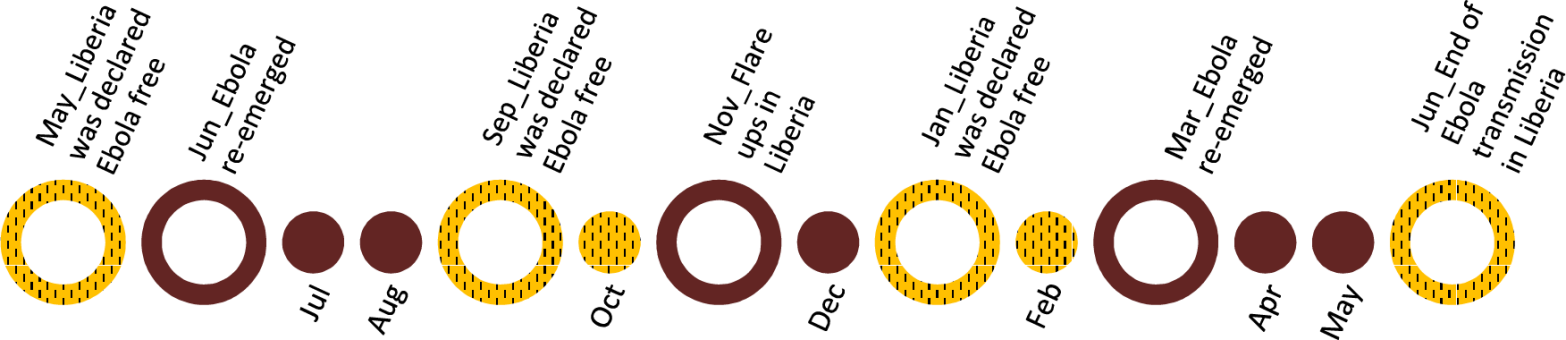
Timeline May 2015 until June 2016 [49].

As visualised in Fig 2, on May 9^th^ 2015 the original Ebola outbreak was declared over and Liberia was first declared free of Ebola transmission. On June 30^th^ 2015 the disease re-emerged, one case was tested positive and succumbed to the disease. On September 3^rd^ 2015 the WHO declared Liberia Ebola free for the second time. Eleven weeks later on November 19^th^ 2015 once again re-infection was reported, with a number of two cases. The 3^rd^ declaration of the end of Ebola transmission was announced on January 14^th^ 2016. New flare-ups were reported starting March 16^th^ 2016, where 8 people died and two survived. Until on June 9^th^ 2016 the end of the EVD outbreak in Liberia was finally declared over [49].

The uncertainty of sexually transmitted flare ups through survivors is reflected in several articles. In November 2015 DOL relates that the transmission routes were obscure:

“Resurgence of the disease has made scientists believe that the Ebola Virus Disease can possibly spread through the semen of a male survivor (for example, by having oral, vaginal, or anal sex). Male survivors are often advised to abstain from sex for a period of 90 days after being discharged from the Ebola Treatment Unit. However, the 90 days sex abstinence is now doubtful as there have been reports of resurgence in Liberia in which the mode of transmission has been linked to sexual transmission in which the survivors had spent over 100 days.

Though it was widely believed that by sexual transmission the male survivor was the donor of the virus through semen, most recent resurgences in Liberia in which the victim was a male has also been linked to sexual transmission. This could mean that the donor in this instance might have been a female.”(B73, 11/24/2015)

The DOL material shows the uncertainty of sexual transmission of Ebola regarding the perceived time span a survivor is still infectious, which is described as between “two to three months” (P70), “the virus is still active in male survivors for 82 days” (A42), to reporting a CDC statement: “contact with semen from male survivors should be avoided! Until research finds out how long the Ebola virus remains in the semen” (B43). Similarly to the latter precautious measures the head of the Incident Management System stressed:

“Ebola survivors should consider correct and consistent use of condoms for all sexual acts beyond three months until more information is available” (B30, 03/30/2015)

Up to the end of the observation period it remains unclear how large the infection potential in male survivors is and it is reported that:

“[…] men have their semen checked to know if they might still be able to spread the infection.” (C7, 03/31/2016)

### Stigma reduction

The DOL also reported on stigma reduction measures and general support initiatives for survivors. It was emphasized that in order to minimize stigmatization a reintegration process was particularly important for individuals following isolation or treatment for Ebola (A9). Especially in articles from the year 2014 reintegration, reunion and reunification ceremonies and efforts were highlighted (A1, A2, A2, A9, A18, A12, A20, A30, A45, A51, A59). Later in the epidemic we found fewer reports on reintegration, or the importance of the process, in 2015 there were four articles reporting on that issue (B4, B18, B68, B76). In an article from 15^th^ January 2016 titled “Liberia, Guinea, S/Leone All Ebola-Free” a WHO spokesmen is quoted outlining a comprehensive approach on survivors required:

“We commend the government of Liberia for developing a national policy that will ensure that EVD survivors have access to medical and psychosocial care, screening for persistent virus, as well as counselling and education. This will assist EVD survivors to reintegrate into family and community life, reduce stigma and minimize the risk of Ebola virus transmission.” (C1, 15/01/2016)

Specific slogans targeting survivors were promoted in the Ebola response and reports about campaigns, workshops and billboard ads reoccurred in the DOL articles. These slogans often picked up the issue of stigmatization and linguistic demarcations:

“Stop the Stigma against Ebola Survivors and Ebola is still real.” (A45, A51, B1, B2, B4, B18, B19)

“Do Not Stigmatize Ebola Survivors. They Are Our People, Embrace Them.”(B27)

“Stopping Ebola is everybody’s business” (A48), “Ebola Must Go!! Ebola is Everybody’s Business” (A50)

Furthermore, also different representatives and authorities were cited: “to ensure that […] survivors of the deadly virus are not stigmatized […] cautioned the public against stigmatizing survivors” (A37) and calling upon communities to show tolerance and dignity to Ebola survivors as well as health care workers treating former Ebola patients and members of burial teams (A39):

“Let us reaffirm that all individuals, whether they are Ebola patients, survivors, clinicians, lab specialists, nurses, doctors, burial teams, family members or aid workers should be free from stigma that this, or any other disease, might carry with it, and instead should be treated with dignity and respect.” (A39, 11/17/2014)

“Community dwellers and house owners should embrace survivors who have been fortunate to return from the Ebola Treatment Units (ETUs) and stop frustrating them with stigmatization” (A49, 12/11/2014)

Identification with Ebola survivors through media was also noted in the DOL, e.g. churches (A19), organizations, football associations and individuals announced in the articles that they identified with Ebola survivors (A21, A46, A53, A60, A61, B3). Others also identified with the overall struggle to defeat Ebola and its stigma on survivors (B2) and articles demanded to:

“[…] admonish those involved in stigmatization and rejecting EVD survivors, to stop doing so and accept them as normal people […] they did not buy, pray or request for the disease and they are human beings like ourselves” (A46, 12/08/2014)

The role of news coverage in stigma reduction is already brought up in August 2014, in an article where the head of the Liberian Business Association was cited, who:

“[…] urged reporters to report on the Ebola crisis in a balanced way that areas and individuals affected will not be stigmatized.” (A4, 08/13/2014)

Another stigma reduction measure specified in the DOL was the adoption of the “Health Workers, Infants and Epidemic Survivors Protection Bill” adopted on November 4^th^ 2014, which provides for compensation schemes to epidemic survivors and insurance incentives for health workers (A22). In how far the bill was enforced does not become evident through the articles.

The DOL reported on the one hand, on general support measures; eighty articles (54%) reported on support ranging from a variety of material donations (including food, beddings, etc.) and solidarity packages to radios and solar lanterns (B11), dignity kits (B13) to direct cash dispensing (B5, B70). The latter was not presented without reservations:

“Giving survivor’s money is good, but if they do not properly plan on how to use what they are receiving from donors, it will be an embarrassment for them in the near future” (B70, 10/06/15)

On the other hand, several of the articles pointed to supporting networks and associations which were established and which organized workshops, counselling, psychosocial support (B8, B9, B11, B28, B42, B75, C3), capacity building management seminars (C3) and e.g. counselling manuals “to help people live, love and laugh again after the virus” (B75).

Approaches to counteract factors contributing to stigma were also portrayed as reducing stigma; thereby we found that awareness-raising about transmission, protection and chances of recovery was most prominently put forward (A9, A10, A15, A36, A48, A51, A52, A63) in the battle against stigmatization (A63).

“to improve public understanding on how the virus spreads, and to provide clear information that can be used to reduce the chances of the virus spreading in affected communities as well as to stop stigmatization of Ebola survivors” (A45, 12/01/2014)

“We need to encourage the acceptance of medically cleared survivors and help communities understand the facts about Ebola transmission.” (B41, 05/04/2015)

Some articles cited the importance of Ebola survivor involvement in the fight against the epidemic. Involvement as in donating blood (A25, A28), inclusion in the National Ebola Taskforce, in the recovery plan or as psycho-social counsellors (A10, B84) or through telling their stories, sharing their experience (A37, A63, B26, B35) and helping to fight the epidemic (A2, A6, A46). It is argued that involving survivors may or will lead to de-stigmatization also in terms of countering factors which contribute to stigma:

“[…] encourage survivors to tell their stories and to address fears that they pose a threat to public health. This is an important part of public outreach in fighting misconceptions about the haemorrhagic fever” (A63, 12/31/2014)

The necessity of involvement was voiced both, from the DOL article author and/or from survivors who expressed the wish to be involved in the fight of the epidemic.

The total number of articles lacked a critical appraisal on success of the support and stigma reduction measures, furthermore we could not find reflection on evidence based measures or evaluation results of measures in the articles (A1-A46, B1-B76, C1-C7).

## Discussion

This study analysed the portrayal of Ebola survivors in one of the leading Liberian newspapers, the Liberian Observer. To the authors knowledge this is the first study that analyses media reporting on EVD survivors in the epidemic 2014–2016. We found that reporting was well-balanced and comprehensive in terms of disseminating available medical knowledge and regarding the social situation of Ebola survivors. The challenges that the reports associated with survivorship were primarily suffering from after-effects, social and economic consequences such as living in despair or with little perspective and experiencing psychological distress. Coupled with reported sigma and discrimination against survivors, the Liberian Observer contextualises factors such as untrustworthiness towards international response as well as local health care systems and inconclusive knowledge on cures and transmission routes, which contributed to the predicament. At the same time the newspaper reported on stigma reduction interventions that target survivors and advocate for further support schemes. The analysed articles appeared to have adopted an overall appreciative and empowering attitude towards EVD survivors, their situations and needs. However, critical reporting on or questioning of the effectiveness of interventions was missing. Equally modest was the journalistic scrutiny regarding mode of support and response by government and NGOs.

Journalists have a special responsibility when reporting on health topics because readers may make important decisions based on this information. Globally, the media is one of the most significant sources of health information and media can play a role in delivering health messages to the public and providing a voice for people on their perspective of illness [50].

### How is the situation of Ebola survivors portrayed?

We observed that the Liberian Observer defined survivors beyond biological terms–something that might reflect the actual perception in Liberian society which is expressed by the often cited statement “we are all survivors”. Broadly speaking, we identified two ways of defining a survivor in the media portrayal analyzed–in social and biological terms. The DOL reflects a broader social definition of survivorship and argues that persons who were not tested positive but „survived”the ETU or quarantine measures, or persons who lost a loved one are to be considered survivors. This reflects day-to-day experiences of affected people, families and communities. Being a survivor as portrayed in the DOL is often associated with being a victim. The identity as victims and the broad concept of survivorship, however, do not go uncontested. Some articles call for an empowerment of survivors. Others debate its broad definition because being a biological survivor with a certificate from an ETU entitles a person to support through NGOs and free access to health care. The narrative that survivors should be considered heroes or at least the “social acknowledgment of their status” [19] was supported by the government and several NGOs as an effort to de-stigmatization and was reflected in several campaigns. The heroic narrative is also explicitly used in the DOL reporting, especially to refer to local health care workers, who were particularly affected during and after the epidemic. It can be assumed that an appreciative and empowering definition and attitude towards survivors in the reporting aimed at reaching this group and advocating for them.

The situation of survivors in the DOL was portrayed comprehensively and included physical and mental problems survivors faced, as well as social and economic challenges rarely studied systematically [17, 51–53]. The DOL reports on various different dimensions of survivorship and consequences of having lived through the disease or being highly affected by the epidemic. Almost in an educational-didactical approach the newspaper explains how realities of survivors are experienced by quoting their stories and providing context to their views and needs. Health related after-effects of Ebola virus disease are well documented in scientific literature [12, 54]. However, the social and economic effects of survivorship have rarely been studied and survivors advocate for the improvement of their situation.

### In what way are issues such as stigma and discrimination against Ebola survivors addressed and what factors are identified to contribute to it?

In the reporting of the DOL the topics stigma and discrimination emerge as very important topics, they increase in frequency over the time period studied. We found that the DOL takes a clearly supportive stance towards Ebola survivors and takes their sides. Survivors are given a strong voice and representation in reporting on stigma and how discrimination is experienced and the DOL takes a very clear educational role towards the general public and its readership.

This finding was actually surprising, as this does not seem to reflect what ordinary people believe. The reality on the ground suggests that stigma and discrimination against survivors is taking place widely [19] and that the DOL seems to act as a corrective to what the larger public believes. As the Liberian Observer online newspaper is only available in English it addresses a distinct readership. Based on the latest published data the illiteracy rates in Liberia are at 52,4% [40] which indicates that the readership might not being representative of the total Liberian population. The uniformity of survivors’ support is unusual and clearly different to media reporting on other victims, i.e. suicidal individuals who are often actively stigmatized in media and in the general public [55].

The DOL gave a broad overview what contributed to stigma and discrimination of survivors, e.g. failing communication, fear of contracting the disease in light of uncertain knowledge on transmission routes, absence of a cure and high case fatality rates. During the reporting period transmission became increasingly linked to survivors but the uncertainty of medical knowledge was actually admitted in the reports, yet of course it remained a major challenge in the hazardous situation.

### Which stigma reduction interventions are covered and how?

To reduce stigma, support and reintegration of survivors was often propagated and reported in the DOL. It could accordingly be shown in the research literature that the involvement of communities and families helped the reintegration process of survivors which again reduced stigmatization [3]. The very fact of talking about stigmatization and de-stigmatization campaigns and increasing the visibility of survivors is important and considered to potentially reduce stigma. The DOL broadly reported how individuals and organization of public interest took the sides of survivors and how survivors were helped with goods and psychosocial support. Awareness-raising on the modes of transmission and on how to protect oneself was a prominent issue; engaging survivors in the response was also reported about and portrayed as crucially important to improve the perception as well as the situation of survivors. However, the effectiveness of these interventions was never critically questioned, specifically whether the stigma reduction measures, support and reintegration schemes were appropriate to reduce stigmatization in communities and at the workplace.

The portrayal of stigmatization of survivors resembles what has been reported in scientific studies from earlier EVD outbreaks [53, 56–59]. Approaches that compared stigmatizing attitudes towards people living with HIV and persons who suffered from EVD found striking similarities between the two groups and conclusions are quite similar to what the DOL was actually promoting: i.e., to empower survivors, mobilize opinion leaders in the communities, and disseminate accurate information [60]. However, specifically in the case of infectious diseases it is also important to acknowledge that there is a fine line of what constitutes justified risk behavior for fear of contagion and irrational blaming and shunning of victims [61]. The persistence of Ebola RNA in semen of survivors [62] and minimally symptomatic individuals [21] might add to these biomedical uncertainties.

### Strengths and limitations

To the best knowledge of the authors this is the first analysis of media reporting on the situation of Ebola virus disease survivors. Even though there is acknowledgment that stigma and discrimination regarding EVD survivors has to be addressed [20], in fact very little research has been conducted so far. The greatest strength of this study is that it scrutinizes questions of portrayal and acknowledgment in one of the largest online newspapers in one of the most affected countries of the recent Ebola outbreak. This study points to the portrayal of social aspects of survivorship and its implications on stigma, transmission and the potential for improved public health (emergency) response [32]. One limitation of this study is that it is restricted to the Liberian Observer online newspaper available in English and therefore identifies a specific portrayal in this Ebola epidemic. Based on the latest published data the illiteracy rates in Liberia are at 52,4% [40]. This means that limitations are also evident due to the readership not being representative of the total population. In a survey on mass media access and consumption in Liberia 63% identified radio as the main source of information, and 29% mentioned friends and family [39]. In the face of social media and internet-based data potential for real-time reporting and surveillance and controlling of infectious disease [63] future studies on media reporting on Ebola survivors are recommended to include multiple media sources and types.
